# Mechanisms and determinants of mRNA Lipid Nanoparticle biodistribution: From pharmacokinetics to clinical translation

**DOI:** 10.1002/btm2.70183

**Published:** 2026-10-07

**Authors:** Zakia Alhareth, César López‐Camacho

**Affiliations:** ^1^ The Jenner Institute, Nuffield Department of Medicine University of Oxford Oxford UK

**Keywords:** biodistribution, lipid nanoparticles, mRNA, pharmacokinetics, tissue targeting

## Abstract

Messenger RNA (mRNA) therapeutics formulated in lipid nanoparticles (LNPs) have transformed the field of nucleic acid medicine by enabling transient, programmable protein expression for vaccination, protein replacement, immunotherapy, and genome engineering. Extending their clinical success beyond vaccines requires a mechanistic understanding of where LNP components and mRNA travel, which cells they enter, where cargo is translated, and how each component is cleared. Such biodistribution outcomes are governed by a complex interplay between nanoparticle physicochemical properties, administration route, protein corona formation, host physiology, intracellular trafficking, and degradation. Importantly, the biodistribution of lipid components, intact mRNA, degraded RNA fragments, and translated proteins represents distinct yet interconnected biological processes that should not be interpreted interchangeably. Advances in molecular imaging, quantitative nucleic acid analysis, mass spectrometry, spatial and single‐cell technologies, and pharmacokinetic modeling are improving their resolution across tissues and time. In parallel, rational lipid engineering, selective organ targeting, biodegradable ionizable lipids, ligand‐directed delivery, and artificial intelligence‐assisted design are expanding opportunities for tissue‐specific delivery beyond the liver and lymphoid organs. However, interpretation remains constrained by assay heterogeneity, incomplete understanding of protein corona dynamics and endosomal escape, species‐dependent differences, and the absence of harmonized regulatory standards. This review examines the mechanisms governing mRNA‐LNP disposition, evaluates what current analytical methods detect and miss, synthesizes preclinical and clinical evidence, and considers the strategies needed to achieve predictable, tissue‐selective, and clinically translatable mRNA delivery.

**Translational Impact Statement**

This review highlights how distinguishing tissue exposure to lipid nanoparticles‐associated lipids and mRNA from functional protein expression is essential for predicting efficacy, off‐target effects, and clearance. Integrating complementary analytical methods with mechanistic and computational models will support the rational design and regulatory evaluation of safer, tissue‐selective mRNA therapeutics.

## 1 INTRODUCTION

Messenger RNA (mRNA) vaccines and therapeutics are reshaping modern medicine, offering opportunities to address infectious diseases, inherited disorders, and cancer. By directing cells to produce specific proteins, mRNA enables transient therapeutic activity without requiring permanent genetic modification. The conceptual foundations of this technology were established in the 1990s, when injected plasmid DNA or mRNA was shown to drive protein synthesis in vivo,^1^ and later studies demonstrated that dendritic cells pulsed with mRNA transcribed in vitro could present antigens and elicit immune responses.^2^ These findings established that mRNA could serve as both an antigen template and an adaptable therapeutic payload. Early clinical success, exemplified by the rapid development and deployment of mRNA vaccines during the coronavirus disease 2019 pandemic, has accelerated the development of mRNA applications in oncology, where mRNA constructs can stimulate immune responses against tumor associated antigens.^3^ In parallel, investigational mRNA therapies are advancing for genetic and metabolic disorders through gene supplementation, protein replacement, and other disease modifying strategies. Effective mRNA delivery depends largely on lipid nanoparticles (LNPs), which protect the RNA from enzymatic degradation, promote cellular uptake, and facilitate cytosolic release. The efficiency and anatomical distribution of LNP mediated mRNA delivery are strongly influenced by administration route, particle size, lipid chemistry, and surface composition.^4^ Despite substantial clinical progress, the pharmacokinetics and biodistribution of formulations containing mRNA and LNPs remain incompletely defined.^5^ Fundamental questions concern how these nanoparticles move through the body, which tissues and cell populations they reach, which biomolecules they encounter and interact with, how long the lipid components and mRNA persist, and where the delivered mRNA produces functional protein.^3^, ^6^ Importantly, LNP associated lipids, intact mRNA, RNA degradation products, and encoded protein follow distinct but interconnected spatial and temporal profiles. Detection of one component therefore cannot be assumed to demonstrate the presence or biological activity of another. These distinctions are central to understanding therapeutic efficacy, immune activation, tolerability, and potential off‐target effects.^7^ They become particularly important as mRNA technologies move from predominantly single dose vaccination toward systemic administration and repeated treatment, where residual lipid components, cumulative exposure, and immune responses to formulation excipients may alter clearance and subsequent biodistribution.^8^ Biodistribution research therefore provides both a mechanistic foundation for formulation design and an essential component of translational and regulatory evaluation. Reliable assessment requires complementary methods that distinguish tissue exposure to lipid components from mRNA localization, molecular integrity, intracellular delivery, protein expression, and clearance.^9^, ^10^, ^11^ Regulatory evaluation must similarly account for the transient nature of mRNA, the potentially different persistence of its lipid carrier and metabolites, and the effects of administration route and formulation composition on tissue exposure. This review examines the molecular and physiological determinants of mRNA and LNP disposition, evaluates what current analytical methods measure and what they cannot establish, and synthesizes preclinical and clinical evidence relevant to the development of predictable, tissue selective, and clinically translatable mRNA therapeutics.

## 2 FROM ADMINISTRATION TO FUNCTIONAL DELIVERY: MECHANISMS GOVERNING mRNA‐LNP DISPOSITION

The biodistribution and pharmacokinetics of mRNA‐LNPs are governed by a series of interconnected biological processes that collectively determine tissue exposure, intracellular delivery, protein expression, and clearance.^12^, ^13^, ^14^ Although individual formulations differ, five core pathways are now widely recognized as the principal determinants of mRNA‐LNP disposition: (i) route‐dependent transport and initial tissue distribution, (ii) physicochemical interactions with biological fluids leading to protein corona formation, (iii) receptor‐mediated cellular uptake and organ tropism, (iv) endosomal escape and intracellular trafficking, and (v) metabolic degradation and immune‐mediated clearance (Figure 1). These pathways act sequentially rather than independently, and alterations at any stage influence downstream pharmacokinetics, tissue specificity, therapeutic efficacy, and safety. Importantly, the localization of LNP associated lipids, intact mRNA, and encoded protein represents distinct but related outcomes with different kinetics. Apparent discrepancies among radiolabeling, molecular quantification, imaging, and protein‐based studies can therefore reflect differences in the biological endpoint measured rather than conflicting results.^15^

**FIGURE 1:**
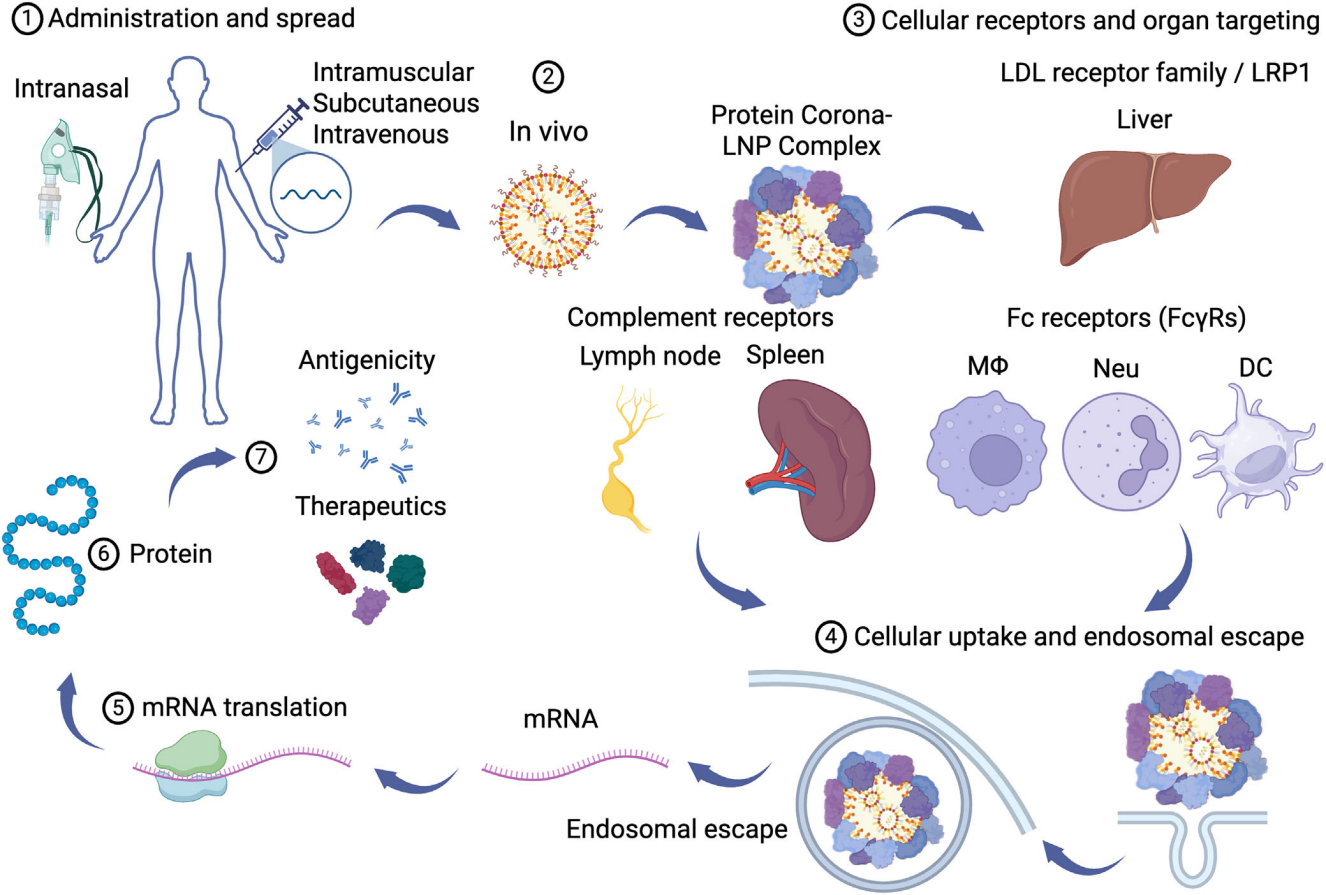
Administration routes, biodistribution, cellular targeting, and intracellular fate of messenger RNA (mRNA)‐lipid nanoparticle (LNP) vaccines. (1) mRNA formulated in LNPs can be administered via multiple routes, including intranasal, intramuscular, subcutaneous, or intravenous injection. (2) Following administration, LNPs distribute in vivo and rapidly acquire a protein corona through interactions with serum proteins. (3) This protein corona influences biodistribution and organ targeting, with prominent uptake for example by the liver via members of the low‐density lipoprotein (LDL) receptor family, and accumulation in secondary lymphoid organs such as lymph nodes and spleen through complement receptor‐mediated interactions. LNPs are also recognized by Fc receptors (FcγRs) expressed on innate immune cells, including macrophages (MΦ), neutrophils (Neu), and dendritic cells (DC). 4) Cellular uptake occurs predominantly via endocytosis, followed by endosomal escape of the LNPs, releasing mRNA into the cytosol. (5) The delivered mRNA is subsequently translated by host ribosomes into (6) the encoded protein antigen, (7) enabling downstream immune recognition and response. Created with BioRender.com.

### 2.1 Route‐dependent tissue access

Following administration, LNPs should undergo rapid distribution through lymphatic and vascular networks, with the extent of dissemination primarily determined by the route of administration, local tissue architecture, and nanoparticle physicochemical properties. Table 1 summarizes representative administration routes, their principal biological barriers, and the distribution patterns reported for each. The discussion below is therefore not an exhaustive catalog, but an examination of routes that illustrate how administration, anatomy, and formulation interact to determine exposure and functional delivery. Intramuscular and subcutaneous administration favor initial retention at the injection site followed by drainage to regional lymph nodes, whereas intravenous administration bypasses local lymphatics and results in immediate systemic exposure with predominant hepatic accumulation. Particle size, surface charge,^3^, ^16^ polyethylene glycol (PEG)‐lipid content, and local vascular permeability further influence transport kinetics and tissue accessibility.^17^ It has been demonstrated that following intramuscular administration, mRNA remained concentrated at the injection site and draining lymph nodes before transient dissemination to the spleen and liver, with peak tissue concentrations occurring within 2–8 h. Subcutaneous administration produced similar distribution but with slower kinetics and prolonged persistence, illustrating that administration route influences both tissue exposure and pharmacokinetic behavior rather than simply changing the magnitude of biodistribution.^18^ Similar observations have subsequently been reported across multiple LNP platforms, confirming that lymphatic drainage is fundamental to vaccine‐induced immunity while systemic exposure remains comparatively limited following local administration.^15^

**TABLE 1:** Conventional and emerging administration routes for messenger RNA (mRNA)‐lipid nanoparticles (LNP) delivery: biodistribution, cellular tropism, pharmacokinetics, and analytical approaches.

Administration route	Initial local exposure	Lymphatic transport	Systemic distribution	Primary target organs/cells	Particle size/formulation	Analytical methods	Proposed mechanism governing biodistribution	Pharmacokinetic characteristics/notes	References
Intramuscular (IM)	High (skeletal muscle, resident APCs)	High; rapid drainage to regional lymph nodes	Low/moderate	Skeletal muscle, draining lymph nodes; low levels in spleen and liver	60–100 nm; conventional ionizable LNPs	RT‐qPCR, IVIS, radiolabeling, flow cytometry, immunofluorescence	Local retention followed by lymphatic drainage to regional lymph nodes, promoting antigen presentation and adaptive immunity	Peak mRNA expression typically 2–8 h; prolonged local expression; limited systemic exposure	17, 18, 19, 20
Subcutaneous (SC)	Moderate–high (subcutaneous tissue, dendritic cells)	High; slower drainage than IM	Low/moderate	Skin, draining lymph nodes; spleen and liver at lower levels	60–100 nm; PEGylated LNPs	RT‐qPCR, fluorescence imaging, flow cytometry	Slower lymphatic drainage than IM, resulting in prolonged local exposure and sustained lymph node delivery	Slower absorption than IM; longer tissue residence; broader lymphatic exposure	14, 18, 21
Intravenous (IV)	Minimal	Minimal (direct vascular delivery)	Very high	Liver, spleen, bone marrow; depending on formulation also lung	70–100 nm; ApoE‐interacting ionizable LNPs	Radiolabeling, LC–MS/MS, MALDI‐MSI, PBPK modeling	Immediate systemic exposure followed by ApoE‐mediated hepatic uptake and mononuclear phagocyte system clearance	Rapid systemic distribution; highest plasma exposure; dominant hepatic clearance	3, 6, 22, 23
Intranasal (IN)	High (nasal epithelium, airway mucosa)	Moderate; cervical lymph nodes	Low	Nasal epithelium, upper respiratory tract, lung	Mucoadhesive or mucus‐penetrating LNPs	IVIS, RT‐qPCR, immunofluorescence	Local mucosal deposition with partial drainage to cervical lymph nodes and induction of mucosal immunity	Rapid local expression; reduced hepatic exposure; potential mucosal immunity	14, 17, 24
Intraperitoneal (IP)	High (peritoneal cavity)	Not specified in source	Not specified in source	Peritoneum, pancreas, liver; peritoneal macrophages, pancreatic β cells	Organ‐selective ionizable LNPs	Bioluminescence, RT‐qPCR, immunohistochemistry	Peritoneal macrophage uptake followed by local transfer to pancreatic tissue; organ capsule filtration contributes to pancreas‐selective delivery	Pancreas‐enriched delivery reported	25
Intrathecal (IT)	High (CSF compartment)	Bypasses BBB; CSF‐mediated distribution	Limited peripheral exposure	Brain, spinal cord; neurons, astrocytes, oligodendrocytes	BBB‐independent LNP formulations; biodegradable ionizable lipids	Fluorescence imaging, RT‐qPCR, immunofluorescence, single‐cell RNA sequencing (scRNA‐seq)	Direct administration into cerebrospinal fluid bypasses the blood–brain barrier, enabling widespread CNS distribution	CNS‐focused delivery route	26, 27
Intratracheal	High (conducting airways)	Not specified in source	Limited systemic exposure	Conducting airways, lung parenchyma; airway epithelial cells, alveolar epithelial cells, alveolar macrophages	Aerosol‐compatible LNPs; biodegradable lipids	In vivo Imaging System (IVIS), confocal microscopy, flow cytometry	Direct airway deposition followed by epithelial uptake and limited systemic exposure	Respiratory delivery route	24
Nebulized/inhaled	High (aerosol deposition in lung)	Not specified in source	Minimal systemic exposure	Distal lung; airway epithelial cells, alveolar macrophages	Aerosolized, mucus‐penetrating LNPs	Whole‐body fluorescence imaging, bronchoalveolar lavage, RT‐qPCR	Aerosol deposition throughout the respiratory tree enables broad pulmonary distribution while minimizing hepatic exposure	Broad pulmonary distribution; reduced hepatic exposure	19, 28, 29, 30, 31, 32
Subretinal	High (local retinal space)	Not applicable/minimal systemic spread	Minimal systemic exposure	Retinal pigment epithelium (RPE), photoreceptors	Biodegradable ionizable LNPs	Histology, immunofluorescence, RT‐qPCR	Direct deposition beneath the retina bypasses inner retinal barriers, enabling efficient local transfection	Local ocular delivery route	33, 34

Abbreviations: ApoE, apolipoprotein E; BBB, blood–brain barrier; CNS, central nervous system; CSF, cerebrospinal fluid; MALDI‐MSI, matrix‐assisted laser desorption ionization mass spectrometry imaging; PBPK, physiologically based pharmacokinetic; scRNA‐seq, single‐cell RNA sequencing.

Regardless of the route of administration, recent studies have shown that PEG‐lipid design is a major determinant of mRNA‐LNP performance rather than a simple stabilizing component. Variations in PEG‐lipid content, acyl‐chain length, and linker chemistry can influence particle stability, circulation time, PEG shedding, cellular uptake, and in vivo potency. In particular, controlled PEG desorption may expose the nanoparticle surface to serum proteins and cell membranes, enabling productive interactions that support tissue uptake and transfection.^35^ Similarly, ionizable lipid architecture, including headgroup composition, linker chemistry, and hydrophobic tail structure, is a major determinant of mRNA‐LNP pharmacokinetics. These features influence protein adsorption, cellular uptake, endosomal escape, lipid metabolism, circulation time, hepatic clearance, and the extent of extrahepatic biodistribution after both intravenous and subcutaneous administration.^36^ Lipid tail chemistry and charge architecture also influence the protein corona, which helps explain why formulations with similar physicochemical size can show different organ tropism and uptake profiles in different biofluids.^37^ For example, quaternary ammonium selective organ targeting (SORT) lipids have been shown to alter protein‐corona composition in parallel with lung‐selective delivery, whereas biofluid‐specific coronas can modify cellular uptake without producing proportional increases in cargo expression.^38^ Collectively, these findings support a mechanistic view in which PEG‐lipid chemistry mainly tunes circulation and stability, ionizable lipid structure governs intracellular delivery and clearance, and tail‐driven corona effects help define tissue tropism.^36^

Respiratory administration enables mRNA‐LNPs to be delivered directly to the airway epithelium, thereby bypassing systemic circulation and first‐pass clearance by the liver. However, it has been noted that intranasal administration is not so straightforward, as animal studies suggest that formulations may be reactogenic. Hence, intranasal, intratracheal or via inhalation delivery, aerosolized or instilled nanoparticles deposit throughout the respiratory tract according to their aerodynamic diameter, with larger particles primarily retained in the upper airways and smaller particles reaching the bronchioles and alveolar regions.^28^, ^29^ Upon deposition, LNPs must first traverse the mucus layer and pulmonary surfactant before interacting with epithelial cells, alveolar macrophages, and resident antigen‐presenting cells.^19^, ^30^ Nanoparticle uptake occurs predominantly through endocytic pathways, including clathrin‐mediated and caveolin‐mediated endocytosis, followed by endosomal escape mediated by ionizable lipids, allowing release of mRNA into the cytoplasm for translation. The locally expressed antigen or therapeutic protein can subsequently stimulate mucosal immune responses, characterized by activation of tissue‐resident dendritic cells, induction of secretory IgA, and generation of tissue‐resident memory T cells, while also eliciting systemic immunity through migration of activated antigen‐presenting cells to draining lymph nodes. Consequently, respiratory delivery not only improves local drug concentration within the lungs but also offers a strategy for inducing robust mucosal immunity against respiratory pathogens while reducing off‐target biodistribution to non‐target organs.^31^, ^32^

Although intravenous administration remains the predominant route for systemic mRNA‐LNP delivery, recent studies demonstrate that intraperitoneal (IP) administration can substantially alter tissue biodistribution and enable pancreas‐selective delivery. It has been shown that ionizable LNPs administered intraperitoneally produced robust mRNA expression in pancreatic β cells through an indirect macrophage‐mediated gene transfer mechanism, whereby peritoneal macrophages internalized LNPs before transferring functional mRNA to pancreatic islets.^25^ This pathway generated efficient pancreatic transfection while largely avoiding the pronounced hepatic tropism typically observed after intravenous administration, highlighting that administration route can fundamentally reshape cellular tropism independently of nanoparticle composition.^25^ More recently, a previously unrecognized mechanism of pancreas‐selective delivery based on organ capsule filtration has been identified. Their pancreatic‐targeted (Arginine‐histidine‐modified LNP (AH‐LNPs) underwent protein‐induced size enlargement following systemic administration, enabling preferential accumulation within the pancreatic capsule before receptor‐mediated uptake by pancreatic cells. The study demonstrated efficient delivery of Cas9 mRNA and therapeutic cytokine mRNAs in rodents and non‐human primates, illustrating that particle size, protein association, organ anatomy and administration route act synergistically to determine organ selectivity, rather than lipid chemistry alone. These findings considerably expand current understanding of mRNA‐LNP biodistribution and suggest that tissue‐specific delivery can be achieved by integrating formulation engineering with anatomical and physiological targeting mechanisms.^39^

Intrathecal (IT) administration has emerged as a promising strategy for achieving efficient mRNA delivery to the central nervous system (CNS) by bypassing the blood–brain barrier (BBB). Unlike intravenous administration, where brain exposure is minimal because of the restrictive nature of the BBB, IT injection introduces LNPs directly into the cerebrospinal fluid (CSF), allowing widespread distribution throughout the brain and spinal cord. This route substantially alters both tissue biodistribution and cellular tropism, enabling direct transfection of neural cells while minimizing peripheral organ exposure. A recent study developed biodegradable brain‐targeting LNPs that achieved widespread mRNA expression following IT administration in mice. Reporter expression was observed across multiple brain regions, including the cortex, hippocampus and cerebellum, with efficient transfection of both neurons and astrocytes, demonstrating that IT delivery can overcome one of the principal barriers limiting systemic mRNA therapeutics. Importantly, the study showed that formulation chemistry remained a critical determinant of CNS biodistribution, with ionizable lipid structure influencing both cellular uptake and regional distribution within the brain.^26^ In addition, it was reported that IT LNPs capable of delivering CRISPR genome‐editing components throughout the CNS. Following administration into the CSF, genome editing was detected in multiple neuronal populations with limited peripheral exposure, highlighting the potential of IT LNPs for treating neurological disorders that require broad CNS distribution.^27^ Also, a review highlighted that optimization of lipid chemistry, particle size, surface functionalization and administration route is essential for overcoming BBB and achieving tissue‐specific CNS delivery.^40^ Consistent with this, a serotonin receptor‐targeted LNPs that increased brain mRNA expression was designed to increase more than 50‐fold following intravenous administration and demonstrated therapeutic efficacy in a glioblastoma model.^41^ Similarly, a furan‐derived ionizable LNPs that exploited meningeal lymphatic transport enhanced brain delivery compared with conventional formulations.^42^ Additional work has shown that optimization of lipid architecture and BBB transport mechanisms further improves neuronal and astrocyte transfection.^43^

Subretinal injection represents an important administration route for ocular mRNA‐LNP therapeutics, particularly for diseases affecting the retinal pigment epithelium (RPE) and photoreceptors. Unlike intravitreal administration, which is limited by the inner limiting membrane and results primarily in transfection of inner retinal layers, subretinal delivery deposits mRNA‐LNPs directly into the subretinal space, placing the formulation near the RPE and photoreceptor cells. This enables efficient local transfection while reducing systemic exposure and has emerged as a promising strategy for the treatment of inherited retinal disorders. Recent studies have demonstrated robust transgene expression and therapeutic efficacy following subretinal administration of mRNA‐LNP formulations, highlighting the profound influence of administration route on tissue distribution, cellular targeting, and therapeutic outcome.^33^, ^34^

### 2.2 Injection‐site laterality and recall responses

An additional factor that may influence mRNA‐LNP biodistribution and immune responses is injection‐site laterality, namely whether sequential vaccine doses are administered in the same limb (ipsilateral) or the opposite limb (contralateral). Emerging evidence suggests that laterality affects lymphatic drainage and recall immunity by determining which draining lymph nodes are re‐engaged during booster vaccination. Ipsilateral boosting preferentially reactivates existing germinal centers, promoting more rapid affinity maturation, T follicular helper cell expansion and early antibody production, whereas contralateral boosting recruits anatomically distinct lymph nodes and may broaden the spatial distribution of germinal center responses. Recent murine studies showed enhanced early germinal center B‐cell responses and higher‐affinity antibodies following ipsilateral mRNA boosting; however, these differences diminished over time, with comparable long‐term antibody titers, neutralizing activity and protection observed irrespective of injection side.^20^ Thus, current evidence indicates that injection‐site laterality primarily influences the kinetics and localization of recall responses rather than the magnitude of long‐term protective immunity. Although clinical evidence remains limited, future biodistribution studies, particularly those investigating repeated mRNA‐LNP administration, should report injection‐site laterality because it may contribute to variability in lymph node targeting, antigen persistence and local reactogenicity.^21^ Beyond lymphatic recall responses, tissue distribution is governed by the interplay between administration route, nanoparticle physicochemical properties and tissue‐specific biological barriers.^22^

### 2.3 From biological identity to functional delivery: protein corona, uptake and intracellular fate

After reaching biological fluids and tissues, mRNA‐LNPs undergo a sequence of extracellular and intracellular interactions that determines whether tissue exposure results in functional protein expression. Protein‐corona formation influences receptor engagement and cellular tropism, but cellular uptake must be followed by efficient endosomal escape and cytosolic release of intact mRNA. Subsequent translation and persistence of the encoded protein determine the magnitude and duration of the biological response, emphasizing that tissue accumulation, cellular uptake, and functional delivery are related but non‐equivalent outcomes.

#### 2.3.1 The protein corona as a biological interface governing organ tropism

Once in biological fluids, mRNA‐LNPs rapidly adsorb plasma proteins to form a protein corona, which replaces the synthetic nanoparticle surface as the biological interface recognized by cells and is now recognized as a major determinant of biodistribution, cellular uptake and clearance rather than a passive coating.^23^, ^37^ Although apolipoprotein E (ApoE) remains the best‐characterized corona component, promoting receptor‐mediated uptake through members of the low‐density lipoprotein receptor (LDLR) family and explaining the intrinsic hepatic tropism of many LNP formulations,^24^ quantitative proteomic studies have demonstrated that the corona is a dynamic mixture of high‐density lipoproteins (HDL), albumin, vitronectin, complement proteins (C3, C4 and C5), immunoglobulins, fibrinogen, prothrombin, C‐reactive protein and α2‐macroglobulin, whose composition depends on lipid chemistry, administration route and host physiology.^44^ These proteins collectively regulate nanoparticle stability, immune recognition, receptor engagement and intracellular trafficking. For example, HDL has been associated with enhanced cellular uptake and transfection efficiency, vitronectin promotes integrin‐mediated interactions, albumin prolongs circulation by improving colloidal stability, whereas complement proteins and immunoglobulins facilitate opsonization and uptake by cells of the mononuclear phagocyte system, accelerating clearance and potentially contributing to complement activation‐related pseudoallergy (CARPA) and anti‐PEG immune responses during repeated dosing.^24^, ^37^, ^45^, ^46^ Similarly, coagulation proteins can influence endothelial interactions and vascular sequestration, while acute‐phase proteins may alter biodistribution under inflammatory conditions.^44^, ^45^ Consequently, formulations with similar physicochemical properties may exhibit markedly different tissue tropism because organ selectivity is determined by the integrated composition of the protein corona rather than ApoE alone. Controlled modulation of the corona through rational lipid design, biodegradable ionizable lipids and SORT strategies has therefore emerged as an effective approach to redirect mRNA‐LNPs from the liver toward extrahepatic tissues, including the spleen, lung and lymphoid organs (Figure 1).^47^, ^48^, ^49^, ^50^ Following receptor‐mediated cellular uptake, productive delivery depends on endosomal escape, the principal intracellular bottleneck for mRNA therapeutics. Ionizable lipids become protonated within the acidic endosomal environment, destabilizing the endosomal membrane and facilitating cytoplasmic release of intact mRNA; however, only a small proportion of internalized nanoparticles successfully escape before lysosomal degradation.^51^, ^52^, ^53^, ^54^ Consequently, enhanced tissue accumulation or cellular uptake does not necessarily result in increased protein expression because intracellular trafficking and endosomal escape ultimately determine functional transfection. Once released into the cytoplasm, mRNA undergoes ribosomal translation before rapid degradation by endogenous ribonucleases, whereas the encoded protein frequently persists considerably longer, creating a temporal dissociation between nanoparticle localization, mRNA persistence and biological activity (Figure 1).^15^, ^55^

#### 2.3.2 Endosomal escape as the bottleneck between uptake and translation

Following tissue localization, LNPs are internalized predominantly through receptor‐mediated endocytosis involving LDL receptors, scavenger receptors, complement receptors, and Fcγ receptors. However, successful cellular uptake alone does not ensure productive delivery. Current evidence consistently identifies endosomal escape as the principal intracellular bottleneck limiting mRNA therapeutics, with only a small fraction of internalized nanoparticles releasing intact mRNA into the cytoplasm before lysosomal degradation. Ionizable lipids facilitate escape by becoming protonated within acidifying endosomes, destabilizing endosomal membranes and promoting cytoplasmic release. Recent in vivo LysoTag and lysosomal barcoding studies have provided the first quantitative measurements of endosomal escape kinetics, demonstrating that relatively small improvements in escape efficiency produce disproportionately large increases in protein expression. These findings shift the focus of formulation optimization from maximizing tissue uptake toward improving intracellular trafficking and cytosolic release.^15^

#### 2.3.3 Translation, protein persistence and clearance kinetics

Once released into the cytoplasm, intact mRNA undergoes ribosomal translation before being degraded by endogenous ribonucleases. Importantly, translated protein frequently persists considerably longer than the mRNA template, creating a temporal disconnect between molecular biodistribution and biological activity. Consequently, biodistribution studies measuring lipid, mRNA, or protein frequently report different pharmacokinetic profiles despite evaluating the same formulation. This distinction is particularly important when comparing radiolabeling, polymerase chain reaction (PCR), and reporter‐based studies, which measure different biological endpoints rather than conflicting aspects of biodistribution.^51^, ^52^, ^53^, ^54^ The final stage of mRNA‐LNP pharmacokinetics is governed by degradation and clearance of both lipid and nucleic acid components. Ionizable lipids are metabolized primarily within the liver and spleen, whereas cholesterol enters endogenous lipid pathways and PEG‐lipids undergo gradual dissociation before hepatobiliary elimination. New biodegradable ionizable lipids containing ester or ketal linkages accelerate hydrolysis, reducing long‐term tissue retention while maintaining efficient delivery. Nevertheless, repeated exposure to PEG‐containing formulations may induce anti‐PEG antibodies and complement activation, leading to accelerated blood clearance (ABC) and altered biodistribution after subsequent administrations. These mechanisms are increasingly relevant as mRNA therapeutics expand beyond single‐dose vaccines toward chronic treatment regimens requiring repeated dosing.^51^, ^56^, ^57^, ^58^, ^59^

## 3 FROM ANIMAL MODELS TO HUMANS: WHAT CURRENT BIODISTRIBUTION EVIDENCE SUPPORTS

Preclinical and clinical evidence together form the foundation of our current understanding of how mRNA‐LNP formulations behave once administered. Early studies in mice, rats, and non‐human primates established that these nanoparticles rarely remain confined to the injection site. Within hours of administration, they travel through lymphatic and vascular networks, distributing to organs of the reticuloendothelial system (Figure 2). In rodents, measurable concentrations of both lipid and mRNA are found in the liver, spleen, and draining lymph nodes, while smaller amounts appear in kidneys, lungs, and occasionally in the heart.^60^ Comparable patterns are observed in non‐human primates, although the magnitude and kinetics of exposure vary according to species differences in lymphatic drainage, metabolic rate, and hepatic clearance.^61^ The liver consistently emerges as the dominant site of LNP deposition and degradation. This tropism reflects the abundance of apolipoproteins and LDLRs that mediate hepatic uptake of nanoparticles through interactions with the protein corona.^45^, ^46^, ^62^ A recent study developed a liver‐targeted, immune‐tolerogenic mRNA‐LNP platform to treat peanut‐induced anaphylaxis. The system delivered mRNA encoding nonallergenic T‐cell epitopes from the dominant peanut allergen Ara h2 to liver sinusoidal endothelial cells (LSECs), key mediators of immune tolerance. Mannose‐modified LNPs enabled targeted delivery, showing strong liver accumulation and increased IL‐10‐producing regulatory T cells in the spleen. In sensitized C3H/HeJ mice, prophylactic treatment alleviated anaphylaxis and suppressed Th2 responses, whereas non‐modified particles showed reduced efficacy. These results demonstrate the potential of mRNA‐LNP platforms for inducing long‐term immune tolerance in food allergy therapy.^63^ The spleen also shows high accumulation, driven by its dense network of macrophages and dendritic cells capable of phagocytosing lipid vesicles and presenting the translated antigen.^61^, ^64^ Lymph nodes act as the principal immunological hub where antigen expression triggers adaptive responses, particularly after intramuscular or subcutaneous administration.^19^, ^65^, ^66^ Expression studies indicate that translation occurs mainly within antigen‐presenting cells and muscle‐resident myocytes, peaking within the first day and declining as mRNA degrades.^17^, ^67^ For example, a study investigated the biodistribution of mRNA‐LNP formulations encoding viral oncoproteins E6 and E7, combined with a TriMix adjuvant containing mRNAs for CD70, CD40L, and constitutively active TLR4 to enhance T‐cell responses. In cynomolgus monkeys receiving two intravenous doses (100 μg each), RT‐qPCR analysis revealed the highest E6/E7 mRNA levels in the spleen, followed by the liver, bone marrow, lymph nodes, and lungs, while levels in the kidneys, brain, heart, and adrenal glands were below detection.^68^

**FIGURE 2:**
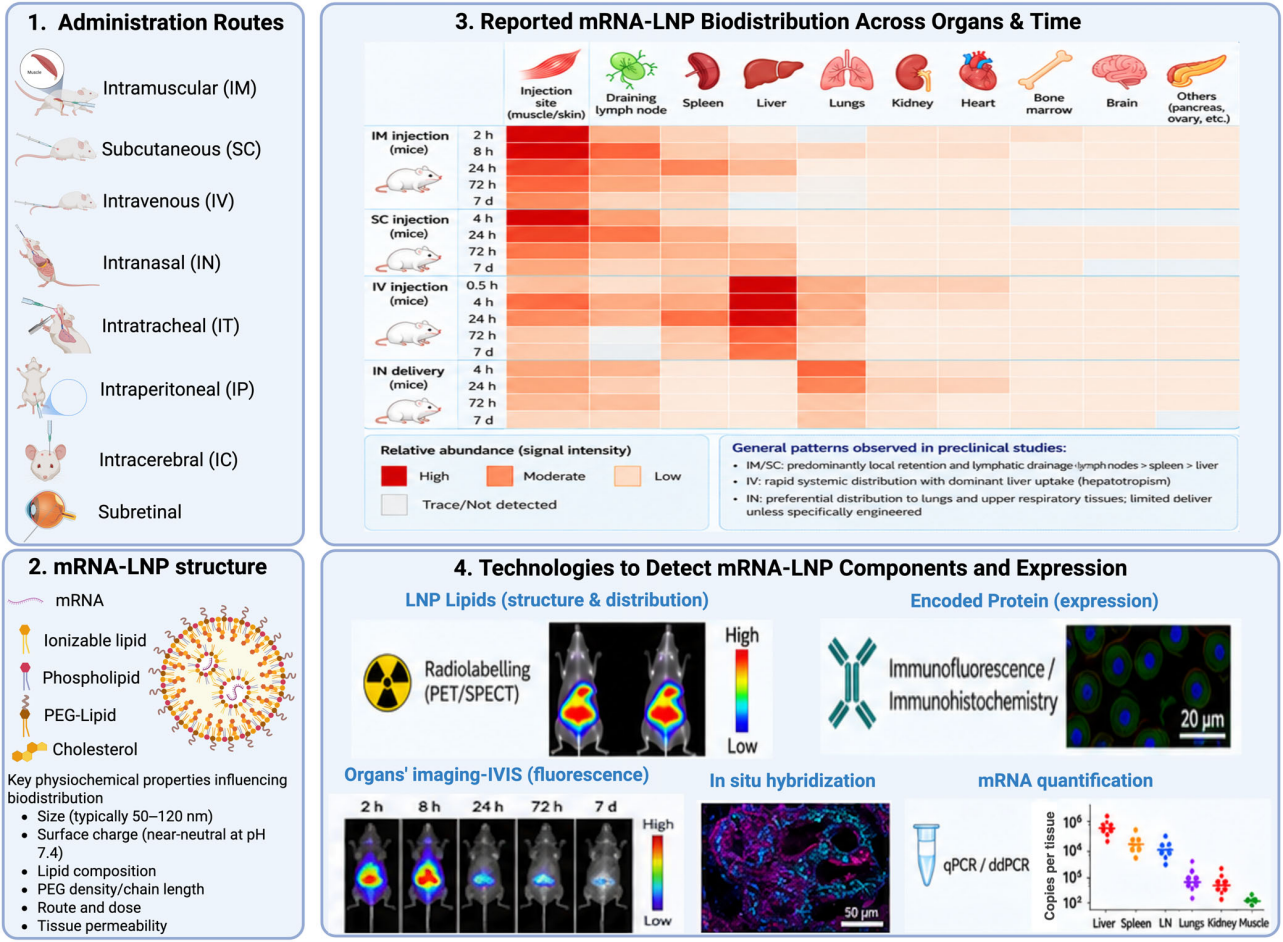
Route‐dependent biodistribution, analytical readouts, and expression kinetics of messenger RNA (mRNA)‐lipid nanoparticle (LNPs). (1) representative intramuscular (IM), subcutaneous (SC), intravenous (IV), and intranasal (IN) administration routes. (2) The general structure of a four‐component LNP comprising an ionizable lipid, helper phospholipid, cholesterol, polyethylene glycol (PEG)‐lipid, and encapsulated mRNA.^69^ (3) Qualitative heat map summarizing reported signal at the injection site, draining lymph nodes, and major organs over time. IM and SC administration generally favor local retention and lymphatic drainage before limited systemic dissemination; IV administration results in rapid systemic exposure with predominant hepatic accumulation; and IN delivery preferentially targets the respiratory tract. Color intensity represents relative signal reported across preclinical studies and is not intended as a quantitative meta‐analysis^17^, ^18^, ^60^, ^61^ (4) Complementary analytical methods used to characterize different stages of mRNA‐LNP disposition. Radiolabeling, PET/SPECT, and fluorescence imaging track labeled LNP components; in situ hybridization and RNAscope localize tissue‐associated RNA sequences; quantitative PCR (qPCR) and digital PCR (dPCR) quantify mRNA‐derived sequences; immunofluorescence and immunohistochemistry detect encoded protein; and bioluminescence imaging monitors functional expression longitudinally. Created with BioRender.com and artificial intelligence tool.

Recent preclinical investigations have clarified how formulation chemistry dictates this pattern. The structure of the ionizable lipid influences both cellular entry and clearance, while the molar ratio of PEG‐lipid controls circulation time and particle stability.^70^ New biodegradable lipids containing ester linkages shorten half‐life and limit long‐term retention in hepatic tissue.^71^ Modulating lipid polarity or introducing zwitterionic components (with a net formal charge of zero, but which contain both positively and negatively charged functional groups) can redirect biodistribution toward the spleen or lung, forming the basis of SORT strategies that now enable extrahepatic delivery.^38^, ^50^ Studies in mice using these formulations have shown up to 10‐fold increases in splenic transfection and improved tolerability under repeat‐dose conditions compared with first‐generation ALC‐0315‐like lipids.^72^

Recent preclinical investigations have demonstrated that small changes in ionizable lipid chemistry can profoundly alter tissue tropism, intracellular delivery and clearance. For example, several biodegradable ionizable lipids following intravenous and subcutaneous administration showed that altering the ionizable lipid head group significantly changed circulation half‐life, hepatic uptake and extrahepatic biodistribution, despite similar particle size and encapsulation efficiency.^70^ Likewise, the PEG‐lipid molar ratio regulates circulation time by controlling PEG shedding; increasing PEG content generally prolongs systemic circulation but may reduce cellular uptake owing to steric hindrance, whereas rapid PEG dissociation facilitates tissue penetration and endosomal delivery.^73^ Biodegradable ionizable lipids containing ester, ketal or acetal linkages have been developed to accelerate metabolic hydrolysis while maintaining efficient mRNA delivery. For example, it was reported that acid‐degradable ionizable lipids that retained transfection efficiency but exhibited significantly faster degradation and reduced hepatic retention compared with conventional persistent lipids, thereby improving repeat‐dose tolerability.^56^ Similarly, a newly developed ketal‐ester ionizable LNPs that reduced hepatotoxicity while enhancing spleen‐selective delivery was produced, demonstrating that rational lipid engineering can simultaneously improve efficacy and safety.^71^

Modifying lipid polarity or incorporating permanently charged SORT lipids has further expanded extrahepatic delivery. A pioneering study demonstrated that inclusion of a fifth SORT lipid redirected mRNA expression from the liver to the spleen or lungs in a charge‐dependent manner, establishing the principle of SORT.^50^ More recently, it was shown that quaternary ammonium SORT lipids promoted lung‐selective mRNA delivery through altered protein corona composition,^38^ whereas it was also reported that splenic B‐cell‐targeting biodegradable LNPs achieved approximately 10‐fold higher splenic transfection than first‐generation ALC‐0315‐like formulations while reducing inflammatory responses following repeated administration.^72^ These studies collectively demonstrate that ionizable lipid structure is not simply a determinant of intracellular delivery but a primary driver of tissue‐specific biodistribution and pharmacokinetic behavior.

Human data, although less comprehensive, is expanding through clinical trials, pharmacokinetic modeling, and post‐marketing analyses of mRNA vaccines. After intramuscular administration, vaccine‐derived mRNA and encoded spike protein can be transiently detected in plasma within the first few hours, consistent with systemic dispersion of a small fraction of the injected dose.^74^, ^75^ Biodistribution studies in lymph node biopsies confirm local expression in germinal‐center B cells and dendritic cells, correlating with the generation of strong antibody and T‐cell responses. Occasional detection of spike antigen in hepatic tissue or in circulating exosomes indicates brief systemic exposure but no sustained expression or accumulation.^76^, ^77^ A study examined the persistence of SARS‐CoV‐2 mRNA vaccines in human tissues and their potential link to cardiac involvement. Using RT‐qPCR, researchers detected vaccine mRNA in lymph nodes, liver, spleen, and myocardium from recently vaccinated individuals. The vaccine was found in axillary lymph nodes of most patients who died within 30 days of vaccination but not in those who died later. In some cases, mRNA was also present in cardiac tissue alongside signs of healing myocardial injury. These findings suggest that vaccine mRNA may persist for up to 30 days and that observed cardiac changes likely reflect pre‐existing conditions rather than direct vaccine effects.^78^

Emerging data suggest that intact LNPs do not readily cross the BBB under normal physiological conditions. Quantitative imaging and molecular assays in non‐human primates have found no evidence of significant mRNA or lipid presence in brain tissue. Similarly, examination of gonadal tissues in vaccinated animals and humans reveals only trace, fragmented RNA species without functional translation, supporting the view that reproductive barriers remain effective. These findings have been corroborated by long‐term safety monitoring of mRNA vaccines, which has not revealed neurotoxic or reproductive adverse effects attributable to biodistribution.^9^

In addition to the BBB, the placenta represents an important physiological barrier that warrants consideration as mRNA‐LNP therapeutics expand into maternal and fetal medicine. Recent studies demonstrate that LNP biodistribution within the placenta is highly formulation‐dependent. Optimized ionizable LNPs can efficiently transfect placental trophoblasts while exhibiting minimal fetal transfer in both pregnant mouse models and ex vivo human placental perfusion systems, suggesting that the placenta can be selectively targeted without substantial transplacental passage under certain conditions.^79^

These findings are significant because they support the development of mRNA therapies for pregnancy‐associated disorders, such as pre‐eclampsia and fetal growth restriction, while also providing reassurance that biodistribution cannot be assumed to equate with fetal exposure. Nevertheless, placental transport is influenced by LNP composition, particle size, PEG‐lipid content, gestational age and route of administration, and systematic evaluation of placental pharmacokinetics remains necessary before widespread clinical application.^80^

The kinetics of degradation and clearance are now better characterized.^81^ In most models, mRNA persists for less than 48 h before completing enzymatic breakdown, while lipid components are metabolized more slowly through hepatic and biliary routes.^82^ PEG‐lipids and cholesterol derivatives may remain for several days, particularly within macrophage populations, but are gradually cleared without significant histopathological changes. Persistence beyond this period is usually associated with incomplete metabolism or tissue sequestration of lipid fragments rather than intact nanoparticles.^83^, ^84^ These insights have highlighted potential implications for repeated administration; pre‐existing anti‐PEG antibodies or residual lipids can accelerate clearance upon re‐exposure, a phenomenon known as ABC.^8^, ^85^ Formulation redesigns that replace PEG with biodegradable hydrophilic polymers are being investigated to mitigate this effect.^85^ Cross‐species comparisons underscore both the robustness and the limitations of existing animal models. While general patterns of hepatic and lymphoid distribution are conserved, quantitative exposure levels vary widely.^86^ Small rodents tend to overestimate hepatic accumulation relative to humans, whereas non‐human primates more accurately replicate human plasma kinetics but differ in immune activation thresholds. Bridging studies that combine ex vivo human tissue models with in vivo animal data are now providing better translational accuracy, particularly for estimating protein expression kinetics and lipid metabolism.^87^ Collectively, the available evidence portrays mRNA‐LNP biodistribution as transient, formulation‐dependent, and primarily confined to lymphoid and reticuloendothelial organs.^9^ Most of the injected dose remains near the injection site or in draining lymph nodes, with a smaller fraction entering circulation and accumulating transiently in liver and spleen before metabolic clearance.^3^ Off‐target deposition in other tissues is minimal under standard dosing, although residual lipid metabolites can persist longer than the mRNA payload itself.^88^ Despite encouraging safety records from billions of vaccine doses, important gaps remain. Few studies have quantified biodistribution after repeated dosing, higher therapeutic loads, or chronic exposure, scenarios that will become relevant as mRNA platforms expand into protein‐replacement and cancer immunotherapy.^82^ Likewise, differences in formulation between commercial and experimental LNPs can yield distinct biodistribution outcomes, complicating cross‐study comparison.^6^, ^60^ Harmonized human studies using standardized analytical platforms, capable of distinguishing intact mRNA from degraded fragments and correlating molecular presence with protein expression, will be essential.^3^ Such efforts will enable precise mapping of tissue exposure, improve the predictive value of preclinical models,^6^, ^61^ and ensure that therapeutic benefit is achieved without compromising long‐term safety.

## 4 CHARACTERIZING THE IN VIVO FATE OF mRNA‐LNPs: METHODS AND INTERPRETIVE LIMITATIONS

Characterizing the in vivo fate of mRNA‐LNPs requires complementary analytical methods that interrogate different components and stages of the delivery process. As summarized in Table 2, these methods differ substantially in sensitivity, specificity, quantitative capability, and spatial and temporal resolution. Measurements of LNP‐associated lipids or labels inform carrier distribution and clearance but do not necessarily demonstrate the presence of intact nanoparticles. Nucleic acid assays localize and quantify targetable RNA sequences, although detection does not by itself establish that the mRNA remains full‐length and translation competent. Conversely, reporter‐ and protein‐based methods demonstrate functional expression but cannot independently determine whether absent or weak expression results from limited tissue exposure, inefficient cellular uptake, RNA degradation, or inadequate endosomal escape. Cell‐resolved imaging, spatial and single‐cell technologies, barcode‐based screening, and endosomal‐escape assays provide additional mechanistic resolution, while physiologically based pharmacokinetic (PBPK) and quantitative systems pharmacology (QSP) models integrate these complementary datasets across tissues, time points, and species. Robust interpretation therefore requires multimodal strategies that distinguish carrier exposure, mRNA localization and integrity, intracellular delivery, and functional protein expression as related but non‐equivalent outcomes.

**TABLE 2:** Comparison of analytical methodologies for investigating messenger RNA (mRNA)‐lipid nanoparticles (LNP) biodistribution and pharmacokinetics.

Methodology	Analyte measured	Spatial resolution	Major advantages	Major limitations	References
Radiolabeling and quantitative whole‐body autoradiography (QWBA)	LNP lipids or radiolabeled mRNA	Whole organ/whole body	Gold standard for ADME studies; highly sensitive; detects total tissue‐associated material independent of protein expression; regulatory benchmark	Cannot distinguish intact LNPs, degraded mRNA or translated protein; requires radioisotopes; specialized facilities; isotope dissociation may affect interpretation	6, 89, 90, 91
Fluorescence imaging (IVIS, DiD, DiR, Cy5/Cy7)	LNP lipid distribution	Whole animal/organ	Non‐invasive longitudinal imaging; rapid formulation screening; real‐time biodistribution	Signal attenuation, tissue scattering, fluorophore dissociation, limited penetration in deep tissues	92, 93, 94
Bioluminescence imaging	Encoded reporter protein (e.g., luciferase)	Whole animal	Measures functional mRNA delivery and protein expression; low background signal	Requires reporter constructs; does not detect untranslated or degraded mRNA; expression depends on endosomal escape	55, 60, 95
RT‐qPCR	Intact mRNA	Tissue level	Highly sensitive; sequence‐specific; widely available	Cannot distinguish intracellular localization or protein expression; amplification bias; detects residual non‐functional RNA	96, 97
Digital PCR (dPCR)	Intact mRNA	Tissue level	Absolute transcript quantification without standard curves; high precision at low copy number	Limited spatial information; higher cost and lower throughput	96, 97
Hybridization chain reaction (HCR)	Intact mRNA	Cellular	Amplification‐free; preserves tissue architecture; visualizes RNA localization	Lower throughput; technically demanding	98, 99
Branched DNA (bDNA)	Intact mRNA	Tissue level	Direct RNA detection without enzymatic amplification; reduced amplification bias	Lower sensitivity than PCR for very low abundance transcripts	100, 101
LC–MS/MS and lipidomics	Ionizable lipids, PEG‐lipids, metabolites	Tissue homogenate	Direct quantification of lipid metabolism and degradation; identifies metabolites	Requires complex extraction and calibration; no cellular localization	6, 102, 103
MALDI mass spectrometry imaging (MALDI‐MSI)	LNP lipids and selected biomolecules	Cellular tissue	Label‐free spatial mapping of lipid distribution; high molecular specificity	Matrix effects; ion suppression; limited quantitative accuracy	103, 104
Flow cytometry/mass cytometry	Cellular LNP uptake; immune phenotype	Single cell	Identifies transfected cell populations; immunophenotyping; high throughput	Requires tissue dissociation; loss of spatial information	61, 105
Immunofluorescence/immunohistochemistry	Encoded protein	Cellular‐subcellular	Visualizes protein expression within tissue architecture; co‐localization with cell markers	Antibody‐dependent; limited quantitative accuracy	60
Spatial transcriptomics and multiplexed In situ hybridization (ISH)	Intact mRNA	Single cell/spatial	Simultaneous molecular and spatial analysis; identifies transfected cell types	High cost; specialized bioinformatics; lower throughput	106, 107
Single‐cell RNA sequencing (scRNA‐seq)	Cellular transcriptome	Single cell	Resolves cell‐specific responses and transfection heterogeneity	Tissue dissociation; expensive; computationally intensive	108
Intravital microscopy	Real‐time nanoparticle trafficking	Cellular (live)	Dynamic visualization of vascular transport, extravasation and cellular interactions	Limited imaging depth; specialized instrumentation	109, 110
PBPK/QSP modeling	Integrated pharmacokinetic parameters	Whole organism	Integrates experimental data to predict tissue exposure, dosing and human translation	Accuracy depends on high‐quality experimental datasets and biological assumptions	111, 112

Abbreviations: ADME, absorption, distribution, metabolism, and clearance; MALDI, matrix‐assisted laser desorption ionization; PBPK, physiologically based pharmacokinetic; PEG, polyethylene glycol; QSP, quantitative systems pharmacology.

### 4.1 LNP distribution and lipid clearance

#### 4.1.1 Radiolabeling and autoradiography

To answer the questions of where the LNP‐associated lipids accumulate, and how they are cleared, radiolabeling remains the reference standard for quantitative biodistribution studies because it enables direct measurement of tissue‐associated nanoparticle components independent of transgene expression. Incorporation of radioisotopes such as ^3^H or ^14^C into lipid components, or ^35^S into the RNA backbone, allows quantitative whole‐body autoradiography, scintillation counting, and tissue radioactivity measurements that provide absolute pharmacokinetic data across all organs over time without relying on successful cellular uptake or protein translation.^89^ Consequently, radiolabeling has been widely adopted during regulatory biodistribution assessments of mRNA‐LNPs and remains the benchmark against which emerging analytical methodologies are compared.^90^, ^113^ The principal strength of radiolabeling lies in its ability to quantify total tissue exposure, enabling robust assessment of absorption, distribution, metabolism, and clearance (ADME). Unlike fluorescence or bioluminescence imaging, which measure reporter activity or optical signal intensity, radiolabeling detects labeled molecules regardless of their biological activity, making it particularly valuable for defining clearance kinetics and identifying organs involved in lipid metabolism. This distinction is important because nanoparticle localization does not necessarily correlate with productive mRNA delivery or protein expression. Several studies have demonstrated that tissues exhibiting high LNP accumulation, particularly the liver, may show relatively modest transgene expression due to inefficient endosomal escape or intracellular degradation, highlighting the importance of interpreting biodistribution and functional delivery as complementary rather than equivalent outcomes.^65^, ^92^, ^93^ Despite these advantages, radiolabeling has important limitations that should be considered when interpreting biodistribution data. Most radiolabeled studies track either the lipid carrier or the nucleic acid cargo individually, rather than simultaneously monitoring lipid distribution, intact mRNA, degraded RNA fragments, and translated protein. Consequently, radiolabeling cannot distinguish whether radioactivity detected within a tissue represents intact nanoparticles, released lipid metabolites, degraded mRNA, or residual labeled fragments. Furthermore, isotope dissociation, radiolytic degradation, isotope‐specific decay correction, and the specialized infrastructure required for handling radioactive materials restrict its widespread application and complicate long‐term studies.^113^, ^114^ These limitations have driven increasing adoption of complementary methodologies, including quantitative PCR (qPCR), digital PCR (dPCR), mass spectrometry imaging, and spatial transcriptomics, which provide molecular or cellular resolution unavailable through radiometric techniques alone.^60^, ^86^, ^92^ An illustrative example is provided by the European Medicines Agency (EMA), 2021, non‐clinical assessment of BNT162b2, in which rats received intramuscular administration of [^3^H]‐labeled LNPs. Quantitative whole‐body autoradiography demonstrated rapid redistribution of LNP‐associated radioactivity from the injection site into systemic circulation, with peak plasma concentrations occurring within 1‐4 h and subsequent accumulation in the liver, adrenal glands, spleen, and ovaries, while approximately 21.5% of the administered radioactivity was recovered in the liver within 48 h.^91^ These findings have frequently been cited as evidence of widespread systemic biodistribution. However, this interpretation should be approached cautiously because the study measured radiolabeled lipid‐associated radioactivity rather than intact mRNA or translated protein. Consequently, these data demonstrate systemic distribution of labeled lipid components but do not establish productive transfection or functional protein expression within those tissues. Subsequent investigations using reporter mRNA, qPCR, and protein expression assays have consistently shown that tissue accumulation and biologically active mRNA expression are only partially correlated, reinforcing the need to integrate multiple analytical platforms when interpreting mRNA‐LNP biodistribution.^6^, ^60^, ^96^ Taken together, radiolabeling remains indispensable for regulatory pharmacokinetic evaluation because of its sensitivity and quantitative accuracy. Nevertheless, it provides only one dimension of the complex biodistribution process. Increasingly, consensus within the field supports combining radiometric techniques with molecular, imaging, and single‐cell approaches to distinguish where nanoparticles localize, where intact mRNA persists, and where functional protein expression ultimately occurs, thereby providing a more complete understanding of mRNA‐LNP disposition and therapeutic activity.^10^, ^96^, ^115^

#### 4.1.2 Optical and bioluminescent imaging

Optical imaging techniques, including fluorescence and bioluminescence imaging, have become indispensable tools for investigating the spatiotemporal behavior of mRNA‐LNPs because they enable non‐invasive, longitudinal monitoring of the same animal over multiple time points, thereby reducing inter‐animal variability and providing dynamic information that cannot be obtained from terminal tissue analyses.^65^, ^92^ Unlike radiolabeling, which quantifies total tissue‐associated radioactivity, optical imaging can distinguish between nanoparticle localization and functional transgene expression depending on the reporter system employed. Consequently, these approaches are particularly valuable during early formulation screening and comparative evaluation of administration routes, where relative differences between formulations are often more informative than absolute tissue concentrations.^92^, ^113^ Fluorescent lipid probes such as Dioctadecyl tetramethylindodicarbocyanine (DiD), Dioctadecyl tetramethyllindotricarbocyanince (DiR), Cy5, and Cy7 are commonly incorporated into the lipid membrane to visualize nanoparticle trafficking and organ distribution, whereas fluorescently labeled nucleotides or reporter mRNAs encoding luciferase or fluorescent proteins (e.g., Green Fluorescent Protein (GFP) or mCherry) enable assessment of intracellular translation following successful cytosolic delivery.^60^, ^93^ Importantly, these reporter systems interrogate different stages of the delivery pathway. Fluorescent lipid dyes primarily reflect the distribution of the lipid carrier, whereas bioluminescent reporters measure functional protein expression, which requires successful cellular uptake, endosomal escape, intact mRNA persistence, and efficient translation. This distinction has become increasingly important because biodistribution of the nanoparticle does not necessarily predict productive mRNA delivery or biological activity. Several studies have demonstrated substantial discrepancies between nanoparticle localization and reporter expression. For example, radiolabeling and fluorescence imaging frequently demonstrate pronounced hepatic accumulation following intravenous administration, yet luciferase expression is often considerably lower than would be predicted from tissue‐associated nanoparticle levels because only a small proportion of internalized LNPs successfully escape the endosomal compartment.^6^, ^55^ Recent investigations have similarly shown that liver macrophages efficiently internalize mRNA‐LNPs but exhibit relatively poor protein expression, illustrating that cellular uptake alone is an unreliable surrogate for therapeutic delivery.^55^ These observations have shifted attention from measuring tissue accumulation alone toward understanding intracellular trafficking and endosomal escape as the principal determinants of transfection efficiency.

Despite these advantages, optical imaging possesses several important limitations. Fluorescence signals are inherently semi‐quantitative and are influenced by tissue depth, optical scattering, absorption by endogenous chromophores, and probe photobleaching, all of which reduce sensitivity in deep organs such as the liver, spleen, and bone marrow.^92^, ^94^, ^113^ Signal intensity may also be affected by fluorophore quenching, dissociation of the fluorescent label from the nanoparticle, or differential degradation of labeled lipid components, potentially leading to overestimation or underestimation of true nanoparticle persistence.^90^, ^113^ Bioluminescence imaging overcomes some of these background limitations because of its high signal‐to‐noise ratio; however, it reports only successful translation of intact mRNA and therefore cannot distinguish whether absent signal reflects poor biodistribution, inefficient cellular uptake, rapid mRNA degradation, or failure of endosomal escape. Consequently, bioluminescence alone provides limited mechanistic insight into the biological barriers responsible for unsuccessful delivery.

These methodological differences explain why optical imaging studies occasionally report biodistribution patterns that differ from those obtained using radiolabeling, qPCR, or mass spectrometry. Rather than representing conflicting findings, these apparent discrepancies often arise because each technique measures different biological endpoints. Radiolabeling quantifies total nanoparticle‐associated material, fluorescence imaging primarily tracks lipid distribution, qPCR measures intact mRNA, whereas bioluminescence reports only functional protein expression. Recognizing these distinctions is essential for accurate interpretation of pharmacokinetic studies and highlights why direct comparison between analytical platforms should be undertaken with caution.^10^, ^96^, ^113^ Consequently, current best practice increasingly favors integrating optical imaging with complementary quantitative approaches, including radiolabeling, dPCR, flow cytometry, spatial transcriptomics, and mass spectrometry imaging, to provide a comprehensive assessment of nanoparticle biodistribution, intracellular trafficking, and functional protein expression. Such multimodal analytical workflows are becoming particularly important as mRNA therapeutics move beyond vaccine applications toward tissue‐targeted therapies, where accurate correlation between nanoparticle localization and biological activity is essential for rational formulation design and regulatory evaluation.^10^, ^95^, ^96^

### 4.2 mRNA localization, abundance, and integrity

#### 4.2.1 Molecular quantification assays

To answer the questions of where the RNA is detected, its amount and its integrity, qPCR and dPCR remain the gold standard for detecting and quantifying mRNA in excised tissues because of their high sensitivity and sequence specificity, enabling assessment of tissue persistence and clearance kinetics even at low transcript abundance.^55^ However, PCR‐based assays detect nucleic acids rather than functional delivery, meaning that positive signals may represent intact mRNA, partially degraded transcripts, or extracellular RNA remnants. Consequently, molecular persistence does not necessarily correlate with protein expression, contributing to discrepancies between PCR‐based studies and imaging or reporter assays.^60^, ^96^ Recent methodological advances, including cap‐specific primers and hydrolysis‐probe assays, have improved discrimination between intact and fragmented mRNA, providing more accurate estimates of biologically relevant transcript persistence.^95^, ^96^, ^97^ Amplification‐free approaches such as hybridization chain reaction (HCR) and branched DNA (bDNA) assays further minimize amplification bias while enabling direct quantification of RNA molecules in tissues.^98^, ^99^ These methods are particularly valuable for distinguishing intact, partially degraded, and translationally inactive mRNA species, thereby improving interpretation of intracellular stability and clearance kinetics.^100^, ^101^ Increasingly, PCR‐based techniques are being integrated with imaging and protein‐based assays to resolve the important distinction between mRNA localization, persistence, and functional protein expression, providing a more comprehensive understanding of mRNA‐LNP biodistribution.^10^, ^96^

#### 4.2.2 Mass spectrometry for lipid and proteomic analysis

Mass spectrometry complements nucleic acid‐based assays by directly quantifying lipid components, lipid metabolites, and translated proteins, thereby providing information that cannot be obtained from PCR or optical imaging alone.^102^ Liquid Chromatography‐mass spectometry (LC–MS/MS) and lipidomic approaches accurately measure intact ionizable lipids, PEG‐lipids, and their degradation products, enabling reconstruction of metabolic pathways and clearance through hepatobiliary and renal systems.^116^, ^117^ Matrix‐assisted laser desorption ionization mass spectrometry imaging (MALDI‐MSI) further localizes LNP lipids within intact tissue sections without requiring radiolabeling, providing valuable spatial information on tissue retention.^102^ Importantly, mass spectrometry has demonstrated that lipid persistence and mRNA persistence are not equivalent, helping explain discrepancies between PCR‐, imaging‐, and radiolabeling‐based studies. While molecular assays often detect intact mRNA for only 24–72 h, lipid metabolites may remain detectable for considerably longer because of slower metabolic clearance, particularly in the liver and spleen.^116^, ^118^ Consequently, studies tracking only lipid components may overestimate the duration of biologically active mRNA delivery if interpreted in isolation. This distinction was illustrated by a quantitative whole‐body autoradiography with LC–MS/MS to investigate the biodistribution of a biodegradable ionizable lipid (“Lipid 5”).^6^ Although lipid‐derived radioactivity rapidly distributed systemically after intravenous administration, LC–MS/MS demonstrated progressive metabolism and elimination through hepatobiliary and renal pathways, with complete clearance by 168 h and minimal long‐term tissue accumulation. These findings highlight the importance of integrating orthogonal analytical techniques, as tissue‐associated lipid does not necessarily indicate persistence of intact mRNA or sustained protein expression. Nevertheless, quantitative interpretation remains dependent on efficient lipid extraction, ion suppression, and appropriate matrix normalization, underscoring the need for standardized analytical workflows when comparing biodistribution studies across laboratories.^103^

### 4.3 Cell‐resolved uptake and functional protein expression

#### 4.3.1 Spatial, single‐cell and barcode technologies

Recent advances in spatial biology have transformed biodistribution studies by resolving which cell populations, rather than simply which organs, receive mRNA‐LNPs. Multiplexed in situ hybridization and spatial transcriptomics localize exogenous mRNA within intact tissue architecture, revealing marked heterogeneity in transfection even within the same organ.^119^ Single‐cell RNA sequencing (scRNA‐seq)^104^ and flow cytometry^120^ further define cell‐specific uptake, intracellular processing, and immune responses, demonstrating that LNP accumulation frequently occurs in phagocytic cells without corresponding productive mRNA translation.^106^, ^107^, ^108^ These observations help explain discrepancies between conventional organ‐level biodistribution studies and functional expression assays, highlighting that tissue exposure alone is an unreliable predictor of therapeutic efficacy.^55^, ^104^ Intravital microscopy complements these approaches by visualizing nanoparticle trafficking through blood and lymphatic vessels in real time, providing mechanistic insight into vascular transport and cellular interactions that static endpoint analyses cannot capture.^106^, ^107^, ^108^ Collectively, these techniques demonstrate that biodistribution is governed by cellular tropism as well as organ tropism, making them increasingly important for the rational design and evaluation of tissue‐targeted mRNA therapeutics.^105^

Recent advances in DNA/RNA barcode technology have transformed the evaluation of mRNA‐LNP biodistribution by enabling high‐throughput, multiplexed in vivo screening of hundreds of nanoparticle formulations within a single animal. Unlike conventional biodistribution studies, which typically assess one formulation at a time, barcode strategies encapsulate unique DNA or RNA oligonucleotide identifiers within individual LNP formulations. Following systemic administration, barcode sequences are recovered from individual tissues or isolated cell populations by next‐generation sequencing, allowing simultaneous quantification of tissue distribution and delivery efficiency across large nanoparticle libraries.^108^, ^109^, ^121^ This approach substantially reduces experimental variability while accelerating the identification of lipid structures associated with tissue‐specific delivery. Barcode technologies have also highlighted an important distinction between nanoparticle biodistribution and functional intracellular delivery. Early studies primarily quantified barcode accumulation within tissues, whereas more recent approaches integrate barcode sequencing with scRNA‐seq or reporter expression to determine which cell types receive functional mRNA delivery rather than simply internalizing nanoparticles.^109^, ^122^ These methods have revealed considerable heterogeneity in delivery efficiency within individual organs and demonstrated that tissue accumulation alone is often a poor predictor of productive transfection. More recently, barcode‐based screening has been combined with LysoTag mice and lysosomal barcoding approaches to quantify endosomal escape in vivo, enabling simultaneous assessment of tissue distribution, intracellular trafficking and functional delivery.^15^

#### 4.3.2 Modeling mRNA‐LNP disposition, protein expression

The increasing complexity of biodistribution datasets has accelerated the adoption of Physiologically based pharmacokinetic models (PBPK) and Quantitative systems pharmacology (QSP) models that integrate imaging, molecular, and lipidomic data to predict absorption, distribution, metabolism, excretion, and protein expression of mRNA‐LNPs.^105^, ^123^, ^124^ These models are particularly valuable because they reconcile datasets generated using different analytical platforms and support model‐informed drug development (MIDD), facilitating dose optimization and translation from preclinical species to humans.^111^, ^125^ However, predictive performance remains constrained by limited high‐quality human biodistribution data, variability in experimental methodologies, and incomplete understanding of key biological processes such as protein corona evolution and endosomal escape.^125^, ^126^ In consequence, discrepancies between model predictions and experimental observations often reflect uncertainties in the underlying biological parameters rather than limitations of the modeling framework itself. As mRNA therapeutics expand beyond single‐dose vaccines toward repeat‐dose and tissue‐targeted applications, integrating standardized experimental datasets with PBPK‐QSP modeling will be essential to improve the accuracy and clinical relevance of biodistribution predictions.^111^, ^126^, ^127^

## 5 TOWARD PREDICTABLE, TISSUE‐SPECIFIC DELIVERY AND CLINICAL TRANSLATION

Controlling biodistribution remains a central challenge in optimizing mRNA‐LNP therapeutics, particularly as these constructs expand into fields such as cancer immunotherapy, protein replacement therapy, and prophylactic vaccination.^11^, ^12^ A key objective is achieving tissue‐specific targeting so that the mRNA payload produces its intended therapeutic or immunizing effect in the correct cellular context while minimizing exposure elsewhere.^48^, ^110^

### 5.1 Next‐generation targeting and biodegradable materials

The ability to guide LNPs selectively toward organs or cell types could transform their therapeutic index and broaden their applicability beyond the liver and lymphoid tissues that dominate current biodistribution profiles.^112^, ^128^ Ligand conjugation has emerged as one of the most promising strategies to achieve targeted delivery. By attaching ligands such as peptides, antibodies, aptamers, or small molecules to the nanoparticle surface, LNPs can be directed toward receptors uniquely expressed on target cells. This approach has shown success in preclinical studies for hepatocyte, endothelial, and tumor‐associated delivery, often improving on‐target mRNA expression by an order of magnitude.^129^, ^130^ Advances in surface chemistry now enable modular ligand attachment without compromising LNP integrity, although challenges remain in controlling ligand orientation, density, and stability during circulation.^130^, ^131^ The formation of a protein corona, whereby serum proteins adsorb onto the LNP surface, can also obscure targeting ligands, highlighting the importance of integrating corona‐resistant coatings into future designs. Equally important is the development of biodegradable LNPs that degrade into non‐toxic metabolites after delivering their cargo.^37^, ^44^, ^132^ New generations of ionizable lipids containing ester or acetal bonds exhibit controlled hydrolysis in physiological conditions, reducing long‐term retention in organs such as liver, spleen, and ovaries. Biodegradability not only accelerates clearance but also mitigates cumulative inflammatory effects observed with repeated dosing. Combining biodegradable lipids with optimized helper lipids and cholesterol analogs has improved tolerability in multiple animal models while maintaining robust mRNA translation efficiency.^71^, ^119^, ^133^ Balancing immune activation and tolerability represents another critical dimension of optimization. Many LNPs act as innate immune stimulants, activating pattern‐recognition receptors such as TLR7, RIG‐I, and NLRP3. While moderate activation contributes to vaccine adjuvanticity, excessive or prolonged stimulation can cause inflammation, hepatotoxicity, or diminished transfection efficiency due to cytokine‐induced translation arrest.^134^, ^135^, ^136^ Rational design of ionizable lipids with lower endosomal escape energy thresholds, combined with partial RNA modification (e.g., pseudo‐uridine substitution), can reduce these effects while preserving immunogenicity where desired. Incorporating selective adjuvant lipids or immunomodulatory peptides also allows tuning of innate responses for different therapeutic goals.^51^, ^137^, ^138^ Dose optimization and control of antigen expression kinetics further refine biodistribution performance.^3^ High doses may saturate phagocytic clearance mechanisms and increase off‐target accumulation in the liver or spleen, whereas subtherapeutic doses yield inconsistent antigen expression.^139^ Controlled‐release LNP formulations and time‐tuned mRNA translation can activate immune cells for a defined duration, limiting tolerance or immune exhaustion.^140^ Quantitative pharmacokinetic models now integrate tissue uptake, lipid degradation, and mRNA half‐life to predict safe and effective dosing intervals. These models are increasingly used to bridge preclinical and human studies and to inform repeat‐dosing regimens for chronic therapies.^141^, ^142^, ^143^ Emerging approaches are beginning to overcome traditional biodistribution limits. SORT nanoparticles exploit charge‐based lipid mixing to redirect mRNA expression toward spleen, lung, or bone marrow^50^; polymer–lipid hybrids and exosome‐mimetic carriers offer improved structural control and compatibility with ligand incorporation^144^; self‐amplifying RNA (saRNA) and circular RNA (circRNA) constructs extend protein expression duration, allowing lower LNP doses and reduced systemic exposure.^114^, ^145^, ^146^ Together, these strategies illustrate a shift from empirical formulation toward rational, data‐driven engineering of biodistribution (Figure 3). Optimization of mRNA‐LNP biodistribution demands an integrated framework that balances four interdependent variables: tissue targeting, clearance kinetics, immune activation, and dose control. Improvements in one dimension often influence the others, reinforcing the need for quantitative, systems‐level analyses that link formulation parameters to biological outcomes. As the field progresses, these principles will underpin the next generation of LNP therapeutics, engineered not only for potency, but also for precision, predictability, and long‐term safety.^11^, ^86^, ^143^, ^147^, ^148^ Advances in nanoparticle engineering are shifting mRNA delivery from empirical formulation toward rational, tissue‐specific design. Polymer‐based nanoparticles, lipid‐polymer hybrid systems and biodegradable ionizable lipids offer improved control over particle stability, degradation kinetics and organ selectivity compared with first‐generation LNPs.^139^, ^149^ In parallel, SORT strategies continue to expand the range of tissues accessible to systemic mRNA delivery, including the lung, spleen, bone marrow and central nervous system (CNS).^44^, ^94^, ^132^

**FIGURE 3:**
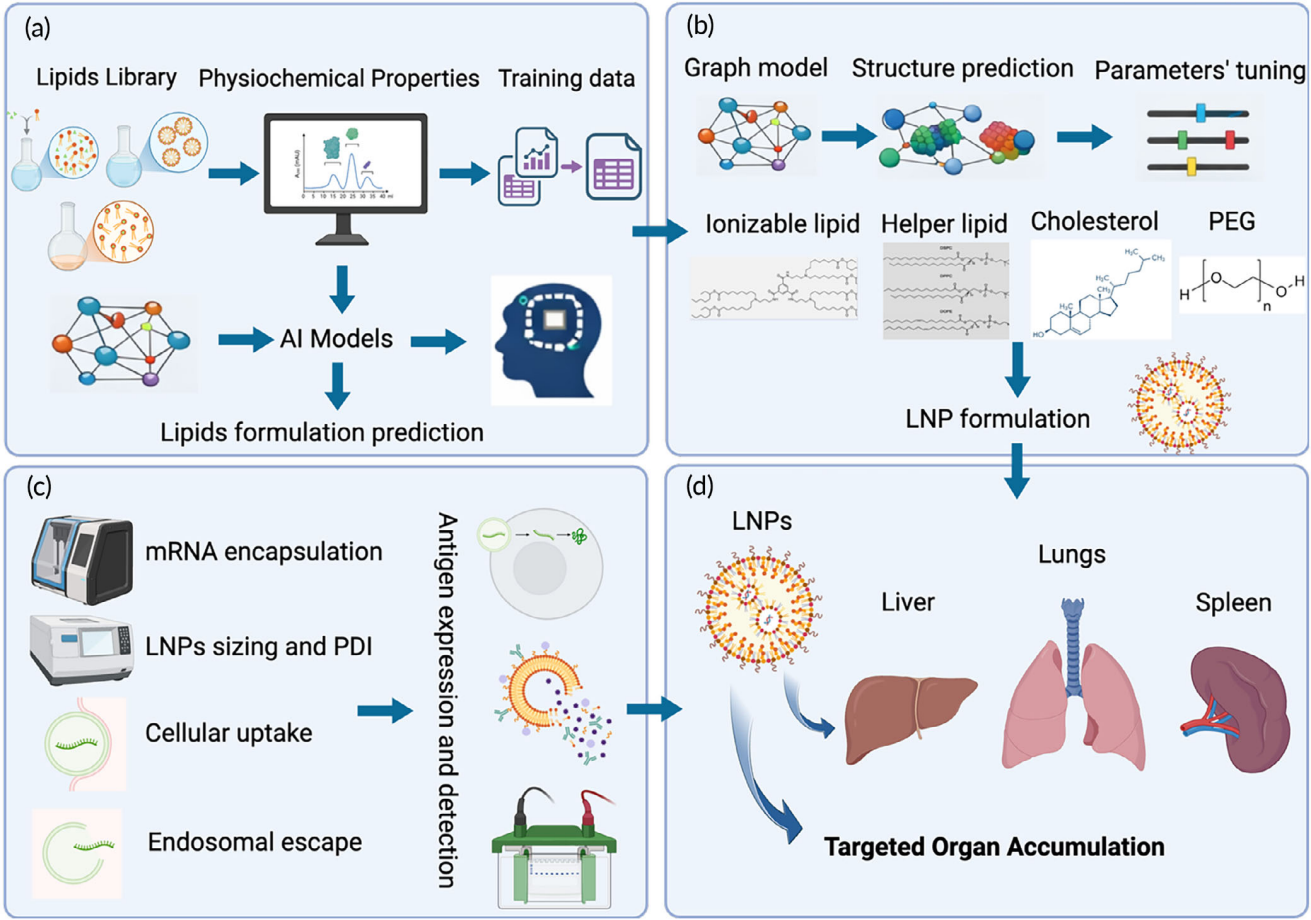
Artificial intelligence‐guided design of lipid nanoparticles for targeted messenger RNA (mRNA) delivery and selective organ targeting (SORT) biodistribution. (A) Machine learning and deep learning models trained on large lipid libraries and formulation datasets predict optimal lipid compositions using physicochemical descriptors and structure–function relationships. (B) In silico optimization, including graph‐based modeling and multi‐parameter algorithms, identifies candidate formulations comprising ionizable lipids, helper lipids, cholesterol, and polyethylene glycol (PEG)‐lipids for efficient mRNA encapsulation. (C) Optimized lipid nanoparticles (LNPs) enhance mRNA delivery, improving encapsulation efficiency, stability, cellular uptake, and endosomal escape. (D) Incorporation of SORT lipids directs tissue‐specific biodistribution, enabling preferential accumulation in organs such as liver, spleen, or lungs.

At the nucleic acid level, saRNA and circRNA platforms extend protein expression while reducing the required RNA dose, thereby decreasing systemic exposure to both mRNA and lipid excipients.^145^, ^149^ These advances may improve safety during repeated administration and broaden the therapeutic applications of RNA medicines beyond vaccination toward chronic protein replacement, regenerative medicine and genome editing.

### 5.2 Bridging species: human‐relevant experimental models

A major challenge in translating mRNA‐LNP therapeutics from preclinical development to clinical practice is the limited predictive value of conventional animal models. Although rodents and non‐human primates remain indispensable for investigating biodistribution, pharmacokinetics and safety, important interspecies differences in lipid metabolism, protein corona composition, immune responses and lymphatic anatomy frequently limit direct extrapolation to humans.^60^, ^61^, ^82^, ^150^, ^151^ Consequently, increasing attention is being directed toward human‐relevant experimental models that better reproduce tissue‐specific physiology. Emerging platforms including organ‐on‐chip systems, three‐dimensional liver and placental organoids, ex vivo human tissue cultures and humanized immune models enable dynamic assessment of nanoparticle transport, cellular uptake and protein expression under physiologically relevant conditions.^152^ These systems are particularly valuable for evaluating tissue‐specific delivery, placental transfer and repeated‐dose toxicity before clinical translation. When integrated with advanced molecular imaging, quantitative pharmacokinetic analyses and computational modeling, they provide an increasingly robust framework for bridging the gap between animal studies and human biodistribution.^61^, ^143^

### 5.3 Artificial intelligence and model‐informed drug development

Artificial intelligence (AI) and machine learning (ML) are emerging as powerful tools for accelerating mRNA‐LNP development (Figure 3), by enabling data‐driven optimization of lipid chemistry, nanoparticle formulation and dosing strategies.^153^ By integrating multidimensional datasets from lipidomics, imaging, transcriptomics and pharmacokinetic studies, ML models can identify relationships between physicochemical properties and biological outcomes, including tissue tropism, transfection efficiency and immune activation.^154^, ^155^ In parallel, generative AI is being used to design novel ionizable lipids, helper lipid combinations and RNA sequence modifications, enabling rapid exploration of formulation spaces that would be impractical using conventional experimental approaches alone.^156^, ^157^ A notable example is a study that developed a graph neural network that combined molecular descriptors, organism‐specific information and curriculum learning to predict BBB permeability of functionalized LNPs. The model successfully identified BBB‐targeting ligands that enhanced neuronal mRNA delivery in vivo, illustrating how AI can prioritize promising formulations, reduce experimental screening and support rational nanoparticle design.^158^ Similar computational approaches have also been applied to predict organ‐specific biodistribution, optimize ionizable lipid structures and integrate formulation design with PBPK modeling, reinforcing the growing role of AI in MIDD.^157^

Despite these advances, the current capabilities of AI should be interpreted cautiously. Most models are trained on relatively small, heterogeneous datasets generated using different formulations, animal models, analytical methods and biological endpoints, limiting reproducibility, external validation and clinical translation.^148^, ^154^ The lack of standardized biodistribution datasets and harmonized reporting further constrains predictive performance, while many deep learning models function as “black boxes,” providing limited mechanistic insight into why specific formulations are predicted to succeed. Moreover, models trained primarily on preclinical data often fail to capture the complexity of human physiology, including protein corona dynamics, immune responses and disease‐specific microenvironments, reducing confidence in their clinical applicability.^146^, ^155^ Future progress will depend on generating large, standardized, high‐quality datasets and integrating AI with mechanistic PBPK and QSP models alongside iterative experimental validation. Rather than replacing laboratory experimentation, AI is more likely to become an integral decision‐support tool that prioritizes candidate formulations, guides experimental design and accelerates optimization while remaining firmly coupled to biological validation and regulatory oversight.^144^, ^152^, ^155^

The future of mRNA therapeutics will increasingly rely on MIDD, integrating PBPK models, QSP, AI and experimental biodistribution data into unified predictive frameworks. Such models enable simulation of absorption, tissue distribution, intracellular trafficking, protein expression and clearance under different dosing regimens, supporting optimization before clinical testing.^143^, ^153^, ^159^ Importantly, these computational frameworks can reconcile data generated by radiolabeling, molecular quantification, imaging and proteomics, improving interpretation of apparently conflicting biodistribution datasets. As more human pharmacokinetic data become available, PBPK‐QSP models are expected to play an increasingly important role in dose selection, repeat‐dose optimization and personalized RNA therapeutics.^127^

Across these analytical approaches, methodological heterogeneity remains a major barrier to cross‐study comparison. Differences in labeling strategy, assay sensitivity, tissue processing, extraction efficiency, calibration, and data normalization can produce divergent results even when similar formulations are evaluated.^115^, ^160^, ^161^ Matrix effects in lipid‐rich tissues and the absence of common reference standards introduce additional uncertainty.^162^, ^163^ Greater harmonization should therefore include transparent reporting of the analyte measured, such as LNP‐associated lipid, intact or fragmented mRNA, or encoded protein; together with assay performance, sampling times, units of normalization, and analytical limits.^164^ Combining standardized, complementary methods will be essential for constructing reproducible maps of mRNA‐LNP distribution, functional delivery, and clearance.^115^, ^165^

## 6 CONCLUSIONS

mRNA‐LNP biodistribution cannot be defined by the localization of a single component or analytical signal. LNP‐associated lipids, intact mRNA, RNA degradation products, and encoded protein follow distinct but interconnected spatial and temporal profiles. Their in vivo behavior is determined by the combined effects of administration route, nanoparticle composition, protein‐corona formation, tissue anatomy, cellular uptake, endosomal escape, translation, and clearance. Consequently, tissue accumulation should not be interpreted as evidence of intact mRNA delivery or functional protein expression. Establishing these relationships will require complementary analytical approaches that distinguish carrier exposure, RNA integrity, cellular delivery, and pharmacological activity across relevant tissues and time points. Advances in lipid engineering, cell‐resolved technologies, barcode screening, endosomal‐escape assays, and predictive modeling are moving the field from empirical formulation toward more mechanistic and model‐informed control of biodistribution. However, the value of these technologies will depend on the quality, comparability, and biological relevance of the data used to develop and validate them.

As mRNA therapeutics expand beyond vaccination into oncology, gene editing, protein replacement, and chronic or repeat‐dose applications, regulatory frameworks must evolve alongside the technology.^10^, ^166^ Existing approaches frequently draw on regulatory principles developed for biologics, which do not fully reflect the component‐specific pharmacokinetics, transient expression, and non‐integrating nature of mRNA‐LNP platforms.^167^ Future evaluations should therefore prioritize validated and complementary biodistribution assays, transparent identification of the analyte measured, appropriate model selection, standardized reporting of tissue exposure and functional expression, and long‐term assessment of lipid metabolites and repeat‐dose effects. AI‐assisted formulation design introduces additional requirements for data provenance, reproducibility, external validation, interpretability, and transparent assessment of model‐derived decisions.^168^ Continued coordination among academia, industry, and regulatory agencies will be essential to ensure that innovation in delivery and computational design is matched by rigorous and proportionate safety evaluation.^169^ Progress toward clinically predictable mRNA therapeutics will ultimately depend on the ability to determine not only where each formulation distributes, but also which cells receive intact cargo, whether functional expression occurs, how long its effects persist, and how these processes influence efficacy and safety.

## AUTHOR CONTRIBUTIONS

**Zakia Alhareth**: Conceptualization; literature investigation; data curation; writing: original draft (lead); visualization; writing: review and editing. **César López‐Camacho**: Conceptualization (Lead); writing: original draft (supporting); supervision; writing: review and editing.

## CONFLICT OF INTEREST STATEMENT

The authors confirm that they have no conflict of interest to declare.

## Data Availability

No new data were generated or analyzed as part of this review. All data discussed are available in the previously published studies cited in the manuscript.
